# Interactions Involving Neutral Surfactants and Double-Stranded DNA: The Case of Polysorbate 80 (Tween 80)

**DOI:** 10.1021/acsomega.5c11135

**Published:** 2026-02-11

**Authors:** Arthur G. S. de Rezende, Márcio S. Rocha

**Affiliations:** Departamento de Física, 28120 Universidade Federal de Viçosa, Viçosa, Minas Gerais 36570-900, Brazil

## Abstract

We investigate the interaction of the neutral surfactant polysorbate 80 (Tween 80) with double-stranded DNA under nearly physiological conditions. Single-molecule force spectroscopy assays performed with optical tweezers were used to determine the binding mechanisms below and above the critical micelle concentration (CMC). Below the CMC, individual surfactant molecules bind cooperatively along the double helix, presenting a high equilibrium association constant on the order of 1 × 10^6^ M^–1^. Above the CMC, on the other hand, micelles can compact DNA at high concentrations (≳140 μM), opening perspectives for novel applications. The present study advances in characterizing DNA interactions with surfactants at the single molecule level.

## Graphical abstract

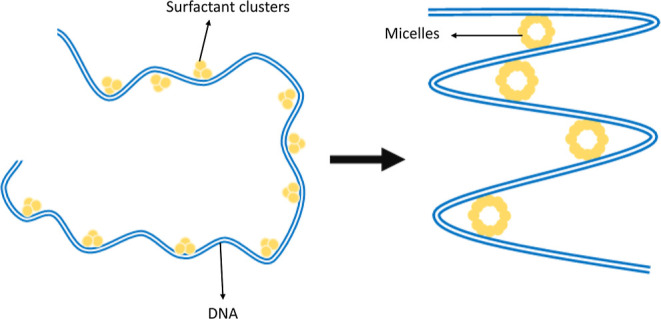

## Introduction

Surfactants are amphiphilic molecules that are well used in a number of industrial applications. In the past years, many researchers have investigated interactions involving diverse types of surfactants and biologically relevant macromolecules.^1–14^ These studies are significant to get information about the toxicity of industrial surfactants, as well as to the development of novel applications for these molecules in processes based on DNA compaction/decompaction mechanisms,^15,16^ purification,^17^ design of new drug delivery carriers,^9^ gene therapies,^6,7,18^ gene expression,^19^ and design of new biosensors,^20^ to cite a few.

An aspect that can drastically influence the interactions involving biomacromolecules and surfactants is the net charge of these molecules in solution. DNA, for instance, is a highly negatively charged biopolymer and tends to interact significantly with cationic ligands, including surfactants.^21^ Recently, we studied the interaction between double-stranded DNA and the monocationic surfactant dodecyltrimethylammonium bromide (DTAB). We found that DTAB molecules first bind individually along the double helix at low concentrations, an interaction mediated mainly by the electrostatic attraction between the negative phosphate backbone of DNA and the positive surfactant head. When a certain surfactant threshold concentration is reached, however, their hydrophobic tails interact significantly to avoid contact with the surrounding water, and such a mechanism starts to dominate the binding process along the double helix, resulting in a strong DNA compaction at higher surfactant concentrations due to the formation of micelle-like bound structures.^14^

In the present study, we turn attention to characterizing the interaction between double-stranded DNA and a neutral surfactant: polysorbate 80, also known as Tween 80, whose chemical structure is represented in Figure 1. We found that, despite being a neutral molecule, PS80 interacts strongly with DNA in a physiologically relevant buffer, presenting an equilibrium constant on the order of 10^6^ M^–1^, which is compatible to the result found for many classic DNA ligands such as drugs and dyes.^21^ To perform such characterization, we use single-molecule force spectroscopy conducted with optical tweezers, which is the state-of-the art technique used to investigate the molecular interactions between biopolymers and ligands.^21^ Besides the binding constant mentioned above, in fact, our approach also allowed the determination of the cooperative character of the interaction, as well as the changes induced on the mechanical parameters of the double helix and the nature of the effective binding mechanism.

**Figure 1:**
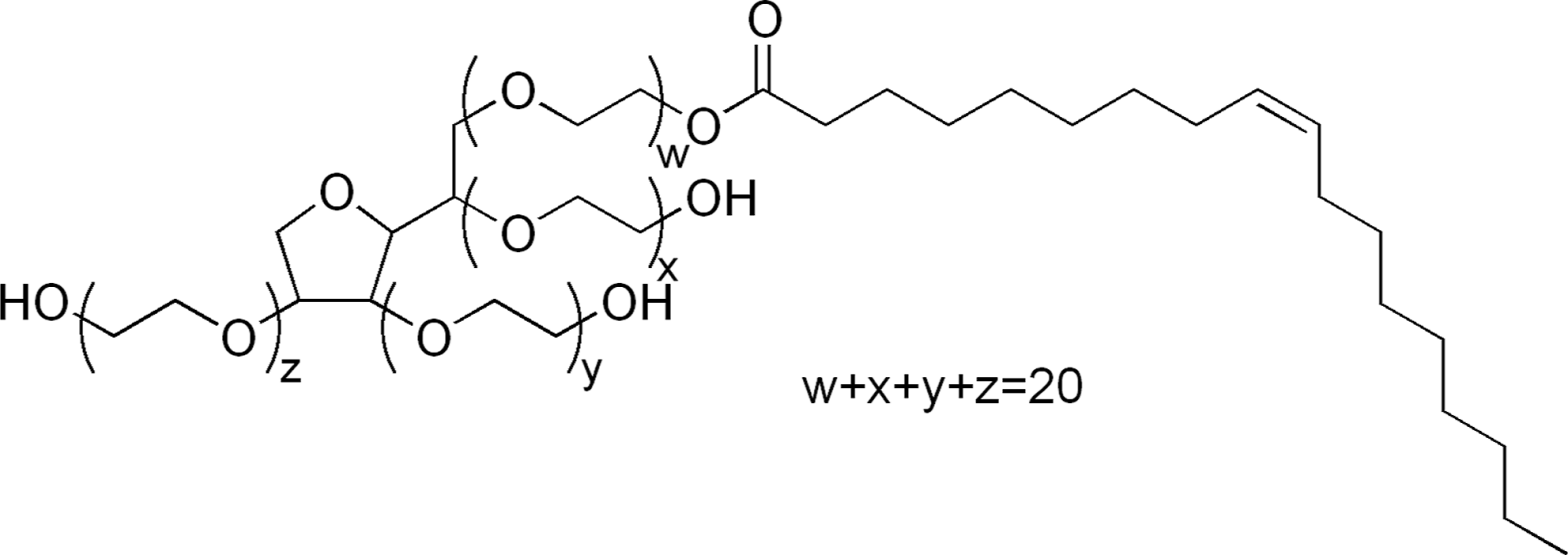
Chemical structure of the surfactant polysorbate 80 (Tween 80), PS80. Note that the surfactant is indeed a heterogeneous mixture with variable degrees of ethoxylation and esterification. Here, we use the specific PS80 from Sigma-Aldrich, Cat. #P8074.

## Materials and Methods

### Sample Preparation and Experimental Procedure for Optical Tweezers Experiments

The samples prepared for the single-molecule force spectroscopy assays consist of a phosphate-buffered saline solution, pH 7.4, containing biotin-labeled λ-DNA molecules tethered by the ends between a 3 μm-sized streptavidin-coated polystyrene bead and a streptavidin-coated microscope coverslip. The solution can be exchanged during the experiments, introducing the surfactant at the desired concentrations. The surfactant solutions at the various concentrations used were prepared immediately before the assays, minimizing aggregation effects. PS80 is weighted and diluted in the same phosphate buffer used to prepare the samples containing DNA.

The optical tweezers used consist of a near-infrared laser (1064 nm) mounted in an inverted microscope, such that one can trap the beads and stretch the tethered DNA molecules using the microscope stage, thus performing the single-molecule force spectroscopy assays to obtain the force–extension curves (FECs) of the bare λ-DNA and its complexes formed with the surfactant PS80 at various different concentrations.

The FECs were collected in the low-force entropic regime (<3 pN) in order to determine the contour and persistence lengths of the complexes with maximum accuracy,^21^ setting the trap stiffness of the tweezers to (3.0 ± 0.1) pN/μm and the pulling speed used to stretch the tethered molecules to 100 nm/s. To determine the mechanical parameters mentioned above, we fit such curves to the Marko–Siggia worm-like chain (WLC) model,^22^ determining these quantities from such fittings. All experiments were performed at ambient temperature (26 °C).

All the details about these experimental procedures and data analyses were reported in previous works.^21,23^

### Determining the Binding Parameters

To determine the effective binding parameters from the persistence length data, we use a quenched-disorder statistical model previously developed in our group to fit the experimental data.^21,24^ Briefly, this model states that the effective persistence length *A* of a DNA-ligand complex can be written in a very general way as a function of the bare DNA persistence length *A*_0_ and two local persistence lengths *A*_1_ and *A*_2_ by the equation

1 $$\frac{1}{A}=\frac{1}{A_{0}}+\left(\frac{2}{A_{1}}-\frac{2}{A_{0}}\right)\frac{r}{r_{\max}}+\left(\frac{1}{A_{0}}-\frac{2}{A_{1}}+\frac{1}{A_{2}}\right){\left(\frac{r}{r_{\max}}\right)}^{2}$$

where *r* is the bound ligand fraction, *r*_max_ is its saturation value, *A*_0_ is the bare DNA (without any ligand) persistence length, *A*_1_ is the local persistence length at a bound site occupied by a single ligand molecule, and *A*_2_ is the local persistence length on the double helix when two bound sites become nearest neighbors.^24,25^

The bound ligand fraction *r* can be connected to an appropriate binding isotherm that describes the interaction effectively. For cooperative binding processes, which is the case for most surfactants,^14^ the Hill isotherm can be used to well describe the physical chemistry of the interaction.^21,25^ Such an isotherm can be written as

2 $$\frac{r}{r_{\max}}=\frac{{\left[K\left(C_{T}-rC_{bp}\right)\right]}^{n}}{1+{\left[K\left(C_{T}-rC_{bp}\right)\right]}^{n}}$$

where *K* is the association equilibrium binding constant, *C*_T_ is the ligand total concentration in the sample, *C*_bp_ is the DNA base-pair concentration in the sample (calculated in the sample preparation procedure as ∼5 μM and confirmed by UV–vis spectroscopy), and *n* is the Hill exponent, a parameter that accounts for the cooperativity degree of binding reactions.

Equation 2 can be used in eq 1 to fit the experimental data of the persistence length as a function of the surfactant concentration in the sample, allowing one to determine the binding parameters and the local persistence lengths.

## Results and Discussion

In Figure 2, we show some representative FECs for various PS80 concentrations, obtained from the force spectroscopy assays, as explained in the former section. The solid lines are fittings to the WLC model, from where the contour and persistence lengths are extracted. Observe that the model fits very well to our experimental data, allowing the determination of these mechanical parameters with high accuracy. We have not noticed any hysteresis or rupture events on the FECs within the force regime used in this work (entropic regime, forces <3 pN). This fact indicates that the interactions are reversible, or at least any irreversibility cannot be detected under this force regime. This is somewhat expected since all intermolecular forces involved in the PS80-DNA interaction are weak (hydrophobic, van der Waals, etc.).

**Figure 2:**
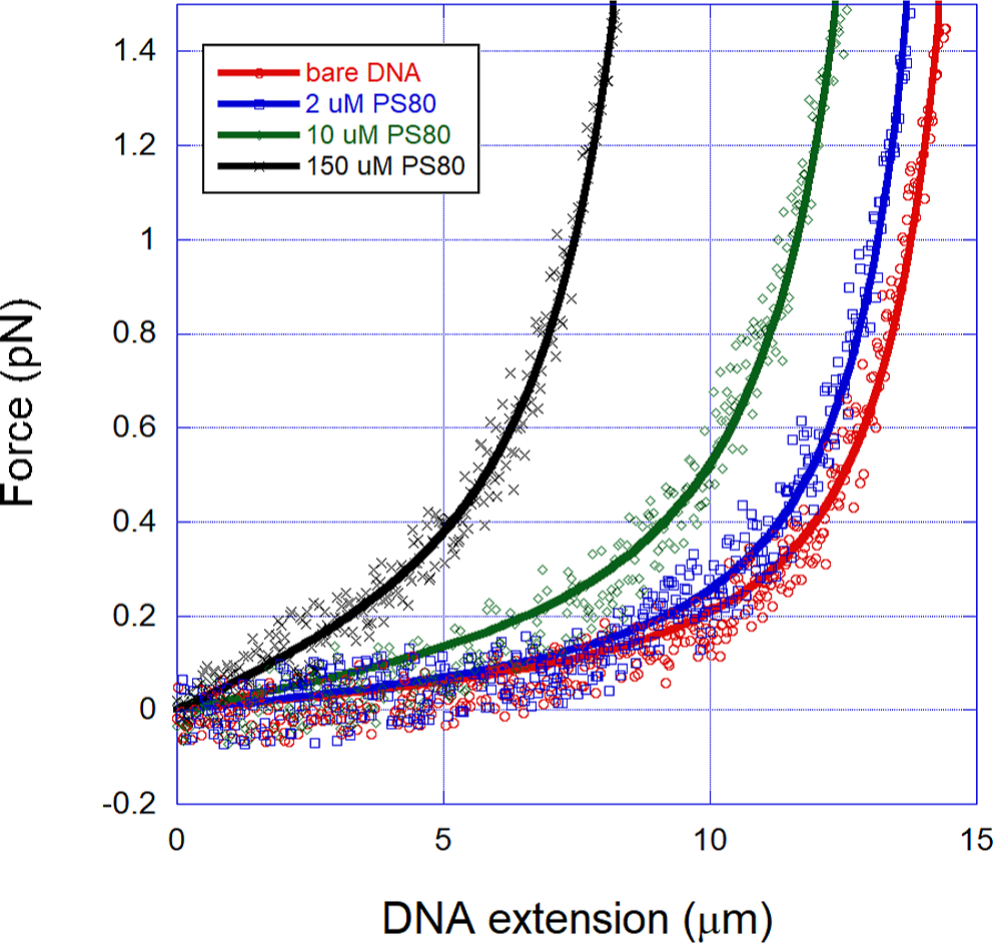
Representative FECs for various PS80 concentrations, obtained from the force spectroscopy assays. The solid lines are fittings to the WLC model, from where the contour and persistence lengths are extracted.

In Figure 3, panel (a), we show the behavior of the contour length of the DNA-PS80 complexes formed as a function of the surfactant concentration in the sample for concentrations below the critical micelle concentration (CMC), which is ∼12 μM for PS80.^26^ Observe that this mechanical parameter remains constant within the error bars and thus cannot be used to infer information about possible binding modes. This result, therefore, indicates that if it really binds to DNA, PS80 does not induce any change in the average interspace between the base-pairs along the double helix. Possible distortions induced by bound surfactant molecules do not contribute here to changing the effective contour length. Thus, we moved to analyze the persistence length, shown in panel (b) of Figure 3 for the same complexes.

**Figure 3:**
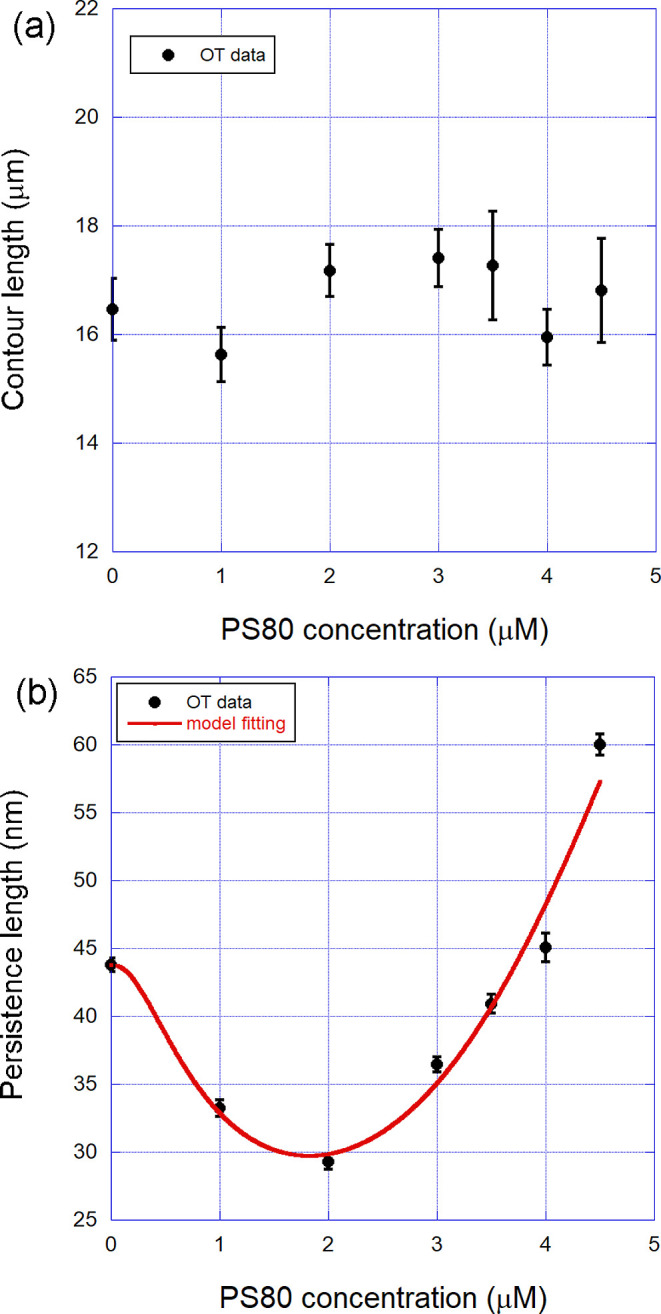
(a) Contour length of the DNA-PS80 complexes formed as a function of the surfactant concentration in the sample, for concentrations below the CMC (∼12 μM). Such result indicates that PS80 does not induce any change on the average interspace between the base-pairs along the double-helix. (b) Persistence length of the same complexes. The nonmonotonic behavior of this mechanical parameter confirms that there is an effective interaction between the surfactant and the double-helix. The red solid line shown in the figure is a fitting to the quenched-disorder model, from where the relevant binding parameters of the interaction were determined. The error bars are the calculated standard error of the mean.

Observe that the persistence length presents an interesting behavior as a function of the surfactant concentration in this range below the CMC, first decreasing for concentrations <2 μM and then increasing for higher concentrations. Such a result confirms that there is an effective interaction between the surfactant and the double helix since the ligand changes the bending rigidity of the double-helix upon binding. The red solid line shown in the figure is a fitting to the quenched-disorder model discussed in the former section and was performed to allow the determination of the relevant binding parameters of the interaction. We found from this fitting the equilibrium binding association constant *K* = (8.3 ± 0.7) × 10^5^ M^–1^ and the Hill exponent *n* = 2.5 ± 0.6; as well as the local persistence lengths *A*_1_ = (16 ± 3) nm and *A*_2_ = (135 ± 10) nm. These parameters are schematically shown in Table 1 for reference. In addition, the parameter *r*_max_ was estimated as ∼0.4, but with a very high error bar (∼60%) returned from the fitting.

**Table 1:** Binding Parameters Obtained from the Optical Tweezers Data for the Complexes Formed between PS80 and Double-Stranded DNA in the Concentration Range below the CMC

*K* (M^–1^)	(8.3 ± 0.7) × 10^5^
*n*	2.5 ± 0.6
*A*_1_ (nm)	16 ± 3
*A*_2_ (nm)	135 ± 10

The behavior of the mechanical parameters (contour and persistence lengths) and the values found for the binding parameters suggest the following possible binding mechanism: our results are consistent with binding in the grooves or along the DNA backbone^21,27–30^ with a high affinity (*K* ∼ 10^6^ M^–1^), a value typically found for many groove binders.^21,25,31^ For comparison purposes, the equilibrium constant found between DNA and the surfactant DTAB (a cationic molecule) under the same experimental conditions is 3 orders of magnitude lower,^14^ a very significant difference that evidences the high affinity between double-stranded DNA and the chemical structure of the PS80 surfactant. Furthermore, the DNA-PS80 interaction is positively cooperative, with a Hill exponent *n* > 1, indicating that the bound surfactant facilitate subsequent binding of new surfactant molecules.^21,25^ Such cooperativity is certainly related to the hydrophobic interactions between the tails of different surfactant molecules, which tend to aggregate, avoiding the surrounding water.^21^ In the present case, such interactions will also involve the DNA base-pairs along the double helix, which are also hydrophobic. Such a mechanism was previously verified for other types of surfactants.^14^ It is also in agreement with the fact that PS80 is known to form premicellar aggregates, with distinct surfactant molecules effectively cooperating, and in this case eventually aggregating on the substrate (DNA). This is fully consistent with the Hill exponent found from the fitting (∼2.5), which indicates that clusters of about 2 to 3 bound surfactant molecules are formed at the binding sites along the double-helix,^24^ as shown in Figure 4. In addition, note that the binding apparently induces local bends that tend to decrease the persistence length, which is compensated later when an excess of bound surfactant is reached along the double helix forming a slightly thicker polymer structure with a higher local persistence length,^32,33^ although persistence length analysis alone cannot discriminate between different binding geometries. In any case, the above competition mechanism can explain the nonmonotonic behavior observed for this mechanical parameter. Figure 4 illustrates this mechanism. Since PS80 is a neutral surfactant, the main intermolecular forces that govern the effective interaction should be van der Waals attraction, hydrophobic interactions involving the surfactant tails and the DNA base-pairs, and others.

**Figure 4:**
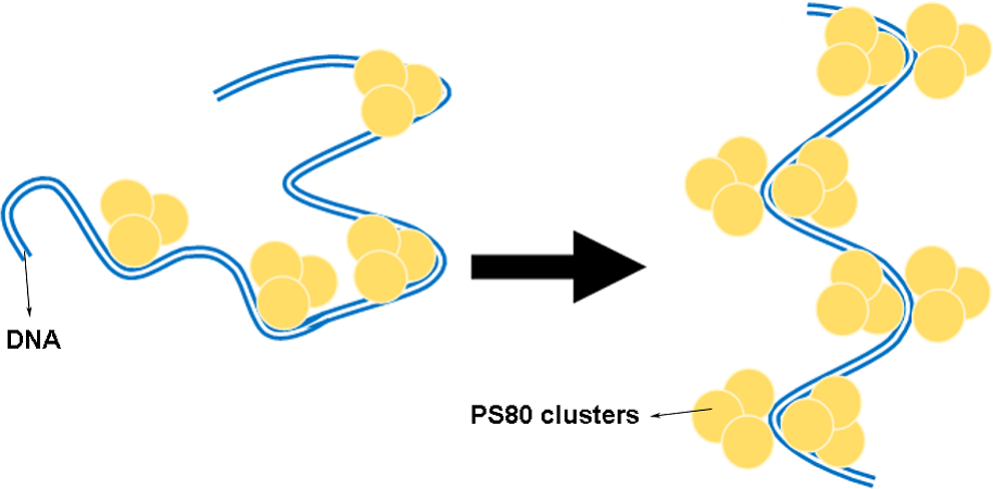
PS80 interaction at the premicellar regime: the surfactant molecules bind to the double-helix inducing bends at the binding sites, decreasing the persistence length. Such effect is compensated later when an excess of bound surfactant is reached, forming a slight thicker polymer structure with a higher local persistence length. This mechanism explains the nonmonotonic behavior observed for this mechanical parameter in Figure 3b.

As mentioned in the Materials and Methods section, the experiments of Figure 3 were performed in a phosphate buffer, with the specific composition is 4.375 mM of Na_2_HPO_4_, 1.25 mM of NaH_2_PO_4_, and 150 mM of NaCl, with a resulting ionic strength of 154 mM. As a second step, the influence of the ionic strength of the surrounding buffer in the binding mechanism of PS80 was also investigated.

In Figure 5, we show the behavior of the mechanical parameters measured for a fixed PS80 concentration (2 μM) for different phosphate buffers prepared using distinct NaCl concentrations in order to regulate the resulting concentration of [Na^+^] in the sample, as described in a previous work.^34^ The point corresponding to the bare DNA molecule, without PS80, is also shown in the figure for reference. Observe in particular that the persistence length for the bare DNA is much higher in very low ionic strengths, a well-established result,^34,35^ but is strongly affected by the binding of PS80. When the surfactant is present, both mechanical parameters remain constant as a function of ionic strength. Observe that we analyzed a very broad range of [Na^+^], from 1 mM up to 220 mM. Such results explicitly show that the interaction between PS80 and double-stranded DNA is basically independent of the ionic strength of the buffer, confirming that the electrostatic character of the interaction is weak as expected for a neutral surfactant. Such a result indicates that the PS80-coated DNA is much less sensible to ionic strengths changes than the bare biopolymer, suggesting that the effective interactions between the surfactant and the double-helix (hydrophobic coating, volume exclusion effect, etc.) reduces ionic sensitivity. In other words, the effects that arise from the interaction likely decrease the effective exposure of the phosphate backbone to the surrounding ionic environment (see Figure 6), thereby reducing the dependence of DNA mechanics on bulk salt concentration—an interesting result that can even find applications in nucleic acids nanotechnology, for example, when one intends to reduce the dependence of these systems on the buffer ionic strength and/or specific composition. Furthermore, coating the double-helix with a relatively large surfactant that presents a high affinity, as in the case of PS80, can also contribute to protecting DNA from degrading agents and enzymes such as nucleases for applications that need this type of protection.

**Figure 5:**
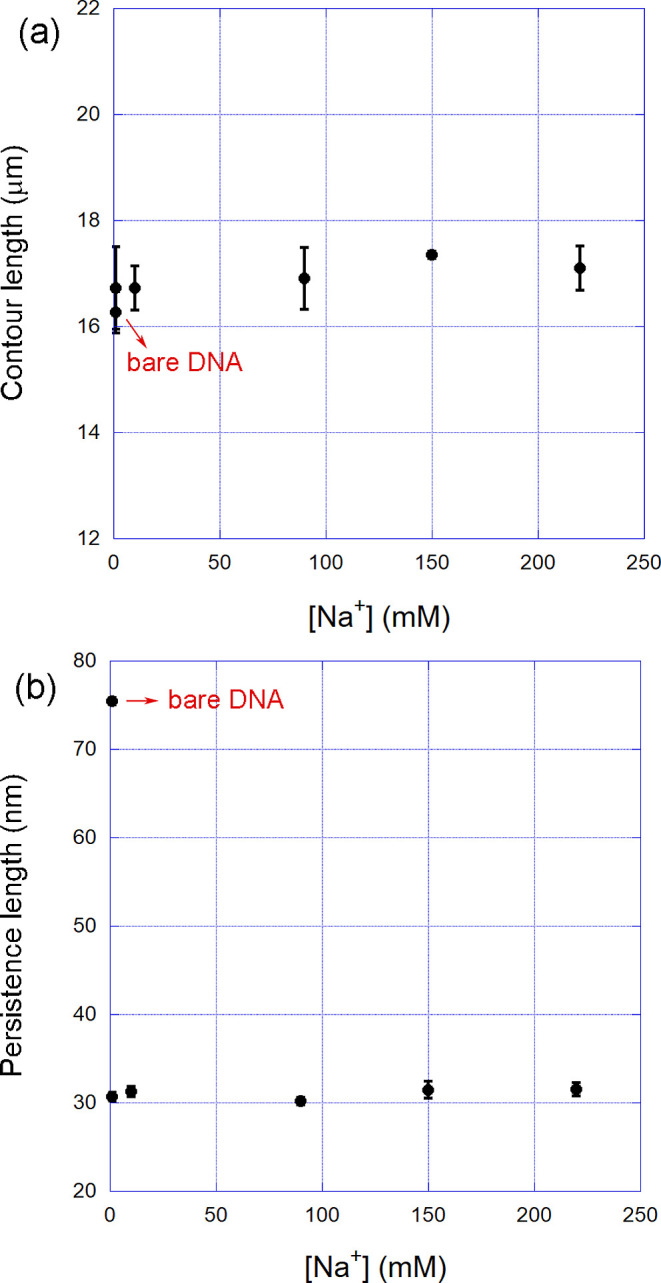
(a) Contour and (b) persistence lengths of DNA-PS80 complexes formed at a fixed surfactant concentration (2 μM) as a function of the [Na^+^] concentration in the sample, i.e., for different ionic strengths. Both mechanical parameters remain constant as a function of the ionic strength, showing that the interaction between PS80 and double-stranded DNA is basically independent of the ionic strength of the buffer (the point corresponding to the bare DNA molecule, without PS80, is also shown in the figure for reference). The error bars are the calculated standard error of the mean.

**Figure 6:**
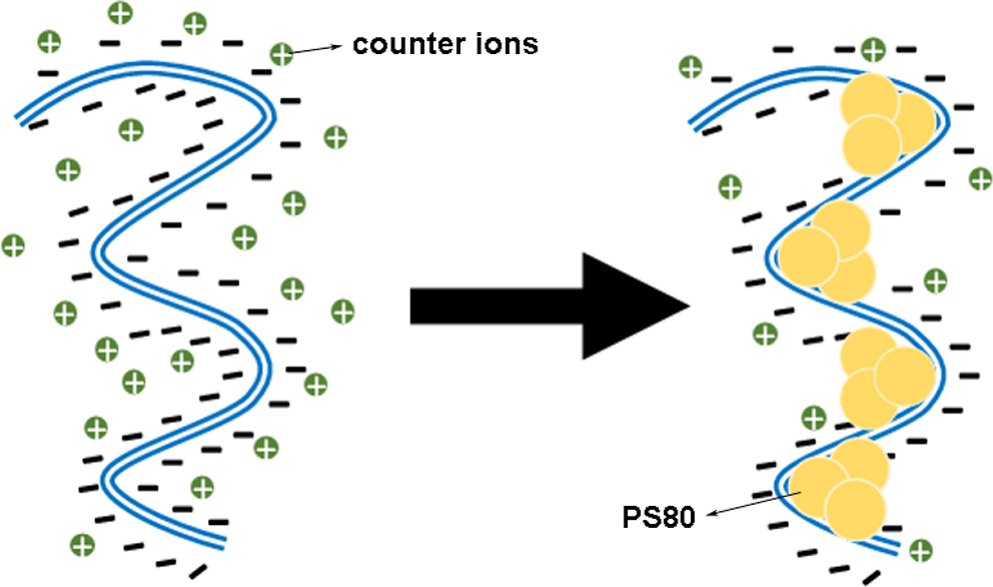
PS80-coated DNA is much less sensible to ionic strength changes than the bare biopolymer, suggesting that the effective interactions between the surfactant and the double helix (hydrophobic coating, volume exclusion effect, etc.) reduce the effective exposure of the phosphate backbone to the surrounding ionic environment, as illustrated.

Finally, the behavior of the mechanical parameters of DNA was also studied in a high concentration range of PS80, well above the CMC. Figure 7 shows the results obtained in these experiments. Observe that the contour length (panel (a)) remains constant within a very broad concentration range but abruptly decays for ≳140 μM. The persistence length (panel (b)), on the other hand, remains practically constant within this concentration range.

**Figure 7:**
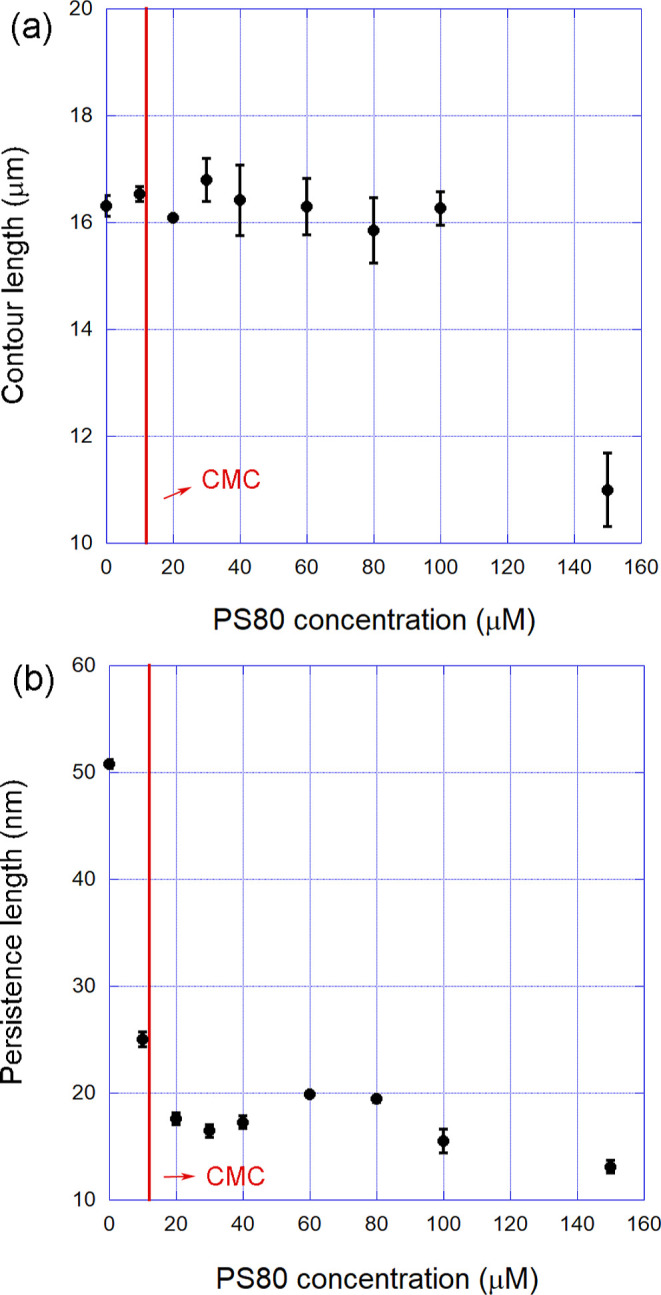
(a) Contour and (b) persistence lengths of DNA-PS80 complexes formed as a function of the surfactant concentration for the range above the CMC (∼12 μM). Observe that the contour length remains constant within a very broad concentration range and then abruptly decays for ≳140 μM. The persistence length, on the other hand, remains practically constant within this range. The error bars are the calculated standard error of the mean.

The result of Figure 7a suggests that PS80 micelles can compact DNA at considerably high concentrations, a result previously verified for other surfactants at the concentration range around and greater than the CMC.^14^ Although such a transition should be studied with more detail in future works, based on previous results verified for other surfactants, it is worth to propose that hydrophobic interactions between micelles and/or premicellar structures bound to the double-helix should be the driving force behind the phenomenon, due to the aggregation between hydrophobic tails from different surfactant molecules localized at distinct binding sites along the double-helix.^14^ Thus, PS80 can find applications in fields in which one intends to compact DNA, such as the development of drug delivery systems. For comparison purposes, while PS80 effectively compacted λ-DNA at concentrations higher than ∼100 μM, the cationic surfactant DTAB can do the same under similar conditions only at a considerably greater concentration, on the order of 400 μM.^14^ Drugs and other small molecules, on the other hand, are usually more efficient, being able to perform such compaction under the same experimental conditions at a concentration range of a few micromolar or less.^36–38^

Finally, it is worth to comment that other nonionic surfactants, such as Triton X-100, have presented a weak capacity to compact DNA, being able to perform such a transition only at so much high concentrations (on the order of 10^1^% weight) via depletion interactions,^39^ a mechanism that cannot be achieved with surfactants in solutions that intend to mimic physiological conditions. Therefore, the fact that PS80 can promote DNA compaction under much lower concentrations is even more interesting and suggests that the specific properties of such surfactant (and similar ones) should be more explored and investigated in the near future. Probably, this is associated with the fact that the chemical structure of PS80 presents a high affinity with DNA even before micelle formation, as shown here, and thus a hydrophobic collapse can occur at higher concentrations, when micelle-like structures dominate the binding sites along the double helix.

In summary, this study advances in characterizing DNA interactions with surfactants at the single-molecule level. In particular, we show that neutral surfactants can interact strongly with the biopolymer both below and above the CMC, presenting specific binding mechanisms and possibilities of novel applications in each case, such as the design of gene therapy vectors,^40^ biosensing platforms,^41,42^ and in DNA compaction technologies,^43,44^ to cite a few.

## Conclusions

The present study advances in characterizing DNA interactions with surfactants at the single-molecule level. We show that the neutral surfactant polysorbate 80 (Tween 80) interacts strongly with double-stranded DNA under nearly physiological conditions. Below the CMC, individual surfactant molecules bind cooperatively along the double helix, presenting a high equilibrium association constant on the order of 10^6^ M^–1^. Above the CMC, micelles can compact DNA for high concentrations (≳140 μM), opening perspectives for novel applications in fields in which one intends to compact the biopolymer for transport purposes.
